# The role of the lateral tarsal strip procedure in modern ophthalmic plastic surgery—A review

**DOI:** 10.3389/fopht.2022.871964

**Published:** 2022-08-26

**Authors:** Adam Kopecký, Alexander C. Rokohl, Ludwig M. Heindl

**Affiliations:** ^1^ Ophthalmology Clinic, University Hospital Ostrava, Ostrava, Czechia; ^2^ Faculty of Medicine and University Hospital Cologne, Department of Ophthalmology, University of Cologne, Cologne, Germany; ^3^ Faculty of Medicine, Department of Craniofacial Surgery, University of Ostrava, Ostrava, Czechia; ^4^ Center for Integrated Oncology (CIO) Aachen – Bonn – Cologne - Duesseldorf, Cologne, Germany

**Keywords:** oculoplastic, eyelid, lateral tarsal strip, ectropion, eyelid surgery, oculoplastic surgery

## Abstract

**Introduction:**

The lateral tarsal strip is one of the basic surgical techniques in ophthalmic plastic surgery. It is used in many indications, predominantly in ectropion repair. Even though there are alternatives, it is probably one of the most popular techniques in ophthalmic plastic surgery. The lateral tarsal strip is also part of bigger surgical procedures (such as midface lifting, entropion surgery, reconstruction surgery, or a part of some surgical approaches to the orbit). The aim of this review is to assess the most common ways of usage of the lateral tarsal strip, to cover its alternatives, and to discuss the future of this technique.

**Methods:**

We have search PubMed and Web of Science and went through articles about lateral tarsal strip. We have also searched for other techniques that used the lateral tarsal strip and included these articles in our review. We have analyzed the major articles and made a review about the topic.

**Results:**

As a natural part of many advanced surgical techniques and as a major surgical technique for lower eyelid ectropion repair, the lateral tarsal strip remains an important part of modern ophthalmic plastic surgery.

## Introduction

The lateral tarsal strip (LTS) procedure is a very popular technique that aims primarily to improve the ectropion in the lower eyelid with increased laxity (1). Nevertheless, as a technique used for a long time, nowadays, its indications and uses are much wider.

This article aims to present the possible indications of the LTS procedure and to discuss its role in modern oculoplastic surgery.

The LTS was firstly introduced in 1979 by Anderson and Gordy (1). From that time, it got widespread popularity, as it was promoted by many prominent ophthalmic plastic surgeons.

There always has been scientific discussion about the indications of the technique, and LTS has been compared to other “traditional” techniques in many studies (2).

Mostly, the LTS was considered equal or superior to many other techniques that aim to improve lower eyelid laxity. The LTS remains widely popular, especially for involutional ectropion and entropion repair.

## Methods

We have searched Web of Science and PubMed. We used the following key word to search for the topic: lateral tarsal strip. We included articles that were focused on the LTS. We excluded articles that described alternative techniques of the surgery without comparison to LTS that was the aim of our article. We also preferred research and review articles to case reports. We have tried to cover most of the important topics that involve modern usage of the LTS. **Table 1** shows the selection of the articles.

**Table 1:** The table shows the process of selecting articles.

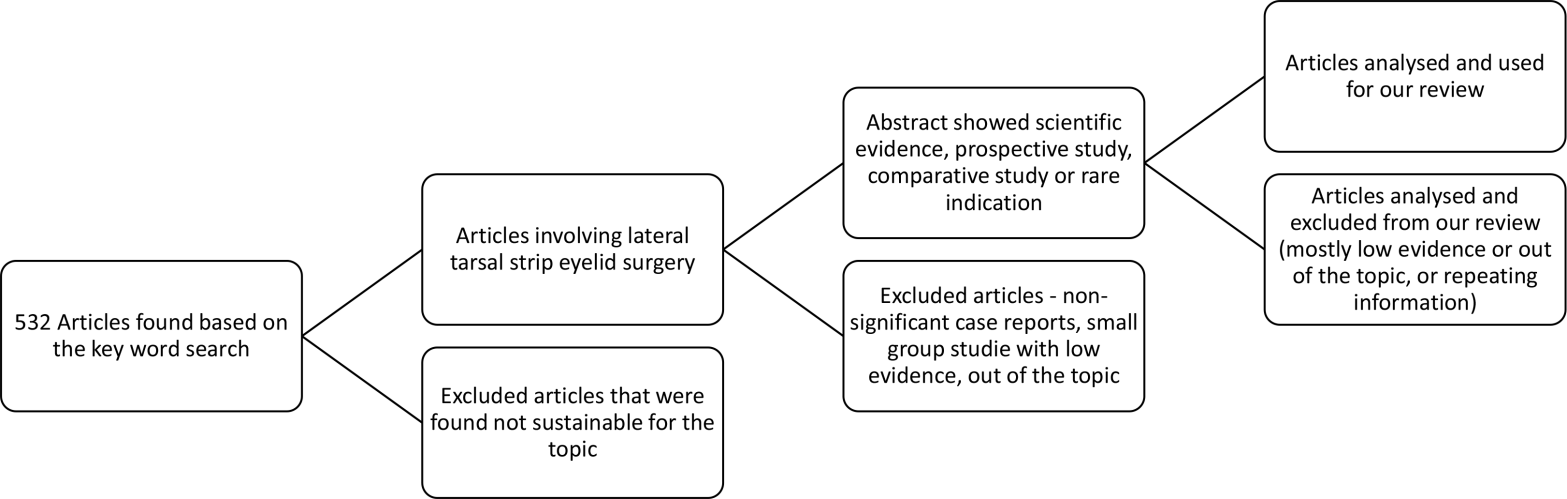

### Technique

**Figure 1:**
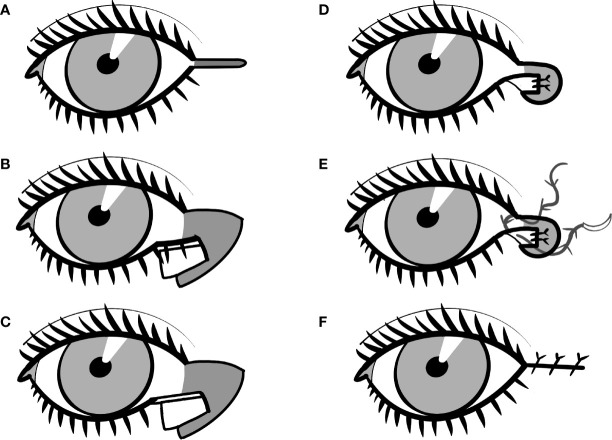
The schematic presentation of the lateral tarsal strip technique. **(A)** A lateral canthotomy and cantholysis and the lower branch of the lateral canthal tendon is separated and the lower eyelid is then fully released from its insertion. **(B)** The lateral portion of the anterior lamella of the lower eyelid is cut. **(C)** The superior portion of the eyelid’s margin is cut off at about the size of the anterior lamella cut. **(D)** The tarsal strip is secured to the periosteum of the medial part of the lateral orbital wall. **(E)** Canthopexy is performed with an absorbable suture. **(F)** The muscle and skin are then closed in two layers.

The LTS is mostly performed under local anesthesia. It begins with a lateral canthotomy and cantholysis (**Figure 1A**). The lower branch of the lateral canthal tendon is separated, and the lower eyelid is then fully released from its insertion.

The lateral portion of the anterior lamella of the lower eyelid is cut (**Figure 1B**), and the superior portion of the eyelid’s margin is cut off at about the size of the anterior lamella cut (**Figure 1C**). The tarsal conjunctiva is cleaned with a scalpel or cauterization, although some authors do not recommend this step as necessary. Now, the so-called “tarsal strip” is prepared for reattachment.

The new position of the eyelid is tested. When more lateral movement of the tarsal strip is needed, lower eyelid retractors are cut with scissors.

The tarsal strip is secured to the periosteum of the medial part of the lateral orbital wall (**Figure 1D**). The surgeon can use either absorbable or permanent sutures. The choice could not be only based on the surgeon’s preference but varies with the primary diagnosis. In most cases, the LTS is sufficient with absorbable sutures (3). Nevertheless, some authors advocate, e.g., in paralytic ectropion to use non-absorbable sutures (4). Again, there is not much evidence in literature to support one type of suture, and the decision is therefore dependent on the surgeon’s preference.

The skin and orbicularis muscle are adjusted to correspond to the new eyelid position. Canthopexy is performed with an absorbable suture (**Figure 1E**). The muscle and skin are then closed in two layers (**Figure 1F**).

Mostly antibiotic ointments are administered after the surgery. The stitches are removed in 7–10 days.

### Possible indications

Indications of LTS include several diagnoses such as involutional ectropion and entropion, paralytic ectropion, and esthetic or even orbital surgery. Some are more frequent, others less; all of them show the wide variety of problems that can be addressed by LTS surgery. In some indications, LTS is only an additional technique in a “bigger” surgical approach; in others, the LTS is the sole technique.

The main advantage of the LTS is the possibility of proper eyelid repositioning and rejuvenation of its lateral fixation (5). Many authors also advocate that, mostly in involutional ectropion, the LTS offers sufficient correction in most cases (6). On the other hand, some studies showed better or the same results with alternative techniques (2, 7). Nevertheless, the LTS remains a technique of choice for many oculoplastic surgeons (8).

Most of the studies of the LTS are based on classic eyelid measurements, patient observations, two-dimensional (2D) photography analysis, or the surgeon’s observations (6, 9, 10). Stereophotography measurements are more and more common in oculoplastic surgery nowadays, and they are proven to be objective, reproducible, and accurate (11–14). These measurements are now standardized with modern investigation protocols that help to perform very objective and reliable measurements (15–17).

As they are works that are using stereophotogrammetry in assessing the lower eyelid and its laxity and tension, more objective research should be presented on this topic to provide definitive arguments for the LTS as a major surgical technique (18, 19).

#### Involutional lower eyelid ectropion

As horizontal eyelid laxity is the major problem of many lower eyelids with ectropion, the LTS is a classic technique that is used in its repair. Involutional ectropion is the major cause of ectropion and it is caused by age, loosening the tension of the orbicularis muscles and both medial and lateral canthal tendons (20).

The success rate of the LTS in this indication is very high (21). The technique is often performed in a standardized manner as written above, and it can be further combined with additional techniques, mostly 3-snip and medial canthoplasty, to affect the medial canthal area as well.

The 3-snip punctoplasty presents a surgical opening (or widening) of the lower eyelid punctum to improve lacrimal drainage (22). Some studies confirm the qualities of the technique (22–24). The technique can be done with scissors or with a punch instrument (25). It is often combined with LTS or done alone.

When medial laxity is also prominent, the LTS can be combined with medial spindle—through the conjunctival incision, the inverting suture is induced to rotate the medial part of the eyelid inward (26). In more severe medial ectropion, the LTS can be combined with a medial canthopexy—from single or two skin incisions, the medial canthus is stabilized with sutures (27, 28).

Even though alternatives to the LTS are often presented in the literature, such as the Bick procedure, the LTS remains the technique of choice in involutional lower eyelid ectropion for most surgeons, as it is easy to perform, highly successful, and with good cosmetic and functional results (6, 21).

#### Involutional lower eyelid entropion

The choice of surgery for lower eyelid entropion depends on its etiology. The LTS positively affects all three major components of involutional entropion: horizontal eyelid laxity, weakening of the eyelid retractors, and override by the orbicularis muscle (29). The loss of tone and laxity are proven consequences of aging (30). Nevertheless, many authors combine the LTS with other procedures for involutional entropion repair.

Commonly, the LTS is combined with lower eyelid retractor plication. It was proven that this combination is more successful than retractor plication alone (31, 32) and that combined surgeries with the LTS are more successful than, e.g., Wies procedure in involutional entropion (33). There is also some experience that these combined surgeries are less prone to recurrence than plication surgeries alone, although further research is needed (34). Another possibility is the combination of the LTS with everting sutures (35). The combined surgery of everting sutures with the LTS has been proven to be more successful than everting sutures alone (36). On the other hand, one study stated that Bick’s procedure with lower lid retractor plication has a lower recurrence rate than the LTS with retractor plication/reinsertion (29).

#### Cicatricial ectropion

The cicatricial cause of ectropion is often more challenging than involutional reasons, since the scar presents strong traction downward that makes the probability of surgical failure higher. The LTS was proven to be useful in managing cicatricial lower eyelid ectropion. In some cases, the LTS itself can provide sufficient correction (37). Mostly, the LTS is combined with another technique—free skin graft, spacer graft for posterior lamella insufficiency, or cheek-midface lifting (38–40).

#### Paralytic ectropion

Several series confirm good results when using the LTS in paralytic ectropion (4, 41). Many advocate the combination of the LTS with lateral (often minimal in size) tarsorrhaphy (42, 43). Surgeons often aim to a higher postoperative position of the lower eyelid in paralytic LTS, as it is presumed that the surgery could be only temporarily successful with some recurrence rate (43). Some papers show that the combination of the LTS with reanimation surgery has higher success in 3-year follow-up (44).

#### Floppy eyelid syndrome

Floppy eyelid syndrome is characterized by high laxity of the upper eyelid that leads to reactive papillary conjunctivitis. It could be seen in patients with sleep apnea, obesity, or keratoconus (45, 46). Initially, it was treated with horizontal eyelid shortening techniques. Nowadays, several modern techniques are used. According to some authors, the LTS in the upper eyelid is considered to be superior to wedge resection techniques (47). Some surgeons recommend combination with medial canthopexy (48).

#### Cheek-midface lift

The LTS is a natural part of the cheek-midface lift technique. The cheek-midface lift is a popular technique for facial rejuvenation in esthetic surgery (49). Also, some reconstruction indications have been described, e.g., for cicatricial ectropion after lower eyelid blepharoplasty or for severe ectropion with a negative vector (50–52).

#### Congenital malpositions

The LTS can also be used in children. Congenital ectropion is very rare, especially the one that is developed because of high lid laxity. But one of the most commonly mentioned indications is congenital euryblepharon. Euryblepharon is characterized by bilateral enlargement of the palpebral fissure with lateral ectropion (53). There are case reports about surgical treatment of epiblepharon with the LTS or with cheek-midface lifting (54).

#### Anophthalmic lower eyelid correction

Proper position of the lower eyelid is important in anophthalmic patients. Loss of volume and higher laxity can lead to malposition of the prosthesis. This leads not only to esthetic concerns of the patients but also to significant discomfort and dry eye (55, 56). Even though this is preventable, sometimes the LTS could aid to improve the fitting of the prosthesis (57). It was even in the first article by Anderson that introduced the LTS as a surgical technique—the initial indication for the LTS was the usage in anophthalmic sockets (1).

#### Esthetic surgery

Canthoplasty is often mentioned in esthetic surgery as stabilization for lower eyelid blepharoplasty (58). However, some surgeons rather combine lower blepharoplasty with the LTS to get better stabilization (59). Microincision is often used to prevent a visible scar in the lateral canthus (60).

With the growing popularity of the transconjunctival approach, it can be presumed that the LTS will be used in lower eyelid blepharoplasty less frequently, as most of the iatrogenic lower eyelid retraction occurs after transcutaneous blepharoplasty (61, 62).

The LTS is often part of the correction of the iatrogenic lower eyelid retraction after excessive blepharoplasty. It is often used with other techniques, such as grafting or cheek-midface lift (39, 50).

#### Tumor resection

Some reports describe the usage of the LTS in the reconstruction of later lower eyelid defects that are in the lateral canthal area. The LTS can be combined with a periosteal flap or even free tarsal grafts and then covered with a myocutaneous transposition flap with very good results (63).

#### Surgical approach to the orbit

In anterior orbitotomy, releasing the lower eyelid from its insertion can play a significant role in getting a better approach to the orbit. It allows the surgeon a better approach to the lower and lateral orbit. Canthotomy or cantholysis is often used in the transconjunctival approach (64). With lateral canthotomy and cantholysis, the surgeon can reach a much wider and deeper space (65). The LTS is then used for repair at the end of the surgery.

## Conclusions

The LTS procedure remains an important basic technique in ophthalmic plastic surgery. Safe, fast, and easy to combine with other techniques, it can be used in a wide range of lower eyelid malposition indications.

## Author contributions

AK - manuscript concept, writing AR - writing, data collection LH - revision of the manuscript, co-writing, clinical analysis. All authors contributed to the article and approved the submitted version.

## Conflict of interest

The authors declare that the research was conducted in the absence of any commercial or financial relationships that could be construed as a potential conflict of interest.

## Publisher’s note

All claims expressed in this article are solely those of the authors and do not necessarily represent those of their affiliated organizations, or those of the publisher, the editors and the reviewers. Any product that may be evaluated in this article, or claim that may be made by its manufacturer, is not guaranteed or endorsed by the publisher.
